# Prevalence and morphological characterizations of *Linguatula serrata* nymphs in camels in Isfahan Province, Iran

**Published:** 2012

**Authors:** Farid Rezaei, Mousa Tavassoli, Moosa Javdani

**Affiliations:** 1 *Department of Pathobiology, Faculty of Veterinary Medicine, Urmia University, Urmia, Iran; *; 2 *Department of Clinical Sciences, Faculty of Veterinary Medicine, Razi University, Kermanshah, Iran.*

**Keywords:** *Linguatula serrate*, Nymph, Camel, Isfahan Province, Iran

## Abstract

*Linguatula serrata*, well known as tongue worm; is an aberrant cosmopolitan parasite, which inhabits the canine respiratory system (final host). The discharged eggs infect many plant feeder animals including human causing visceral and nasopharyngeal linguatulosis which is known as “Marrara syndrome”. In current study, the prevalence of infection with *L. serrata* nymphs in mesenteric lymph nodes (MLNs) of slaughtered camels was investigated in Isfahan Province, Iran. The MLNs of 232 slaughtered camels, including 115 females and 117 males, were examined for *L. serrata* nymphs. Camels were categorized into four age groups, namely under six months, six months to two years, two to four years and greater than four years. Also, the morphometrics of the nymphs were measured using the classic parasitology methods. Results showed that 21.12% of examined camels were infected with *L. serrata*. Age and sex had no significant effect on the prevalence of this parasite in camels. The size of the different parts of nymphs’ body were recorded and evaluated. The infection rate to the nymphs of parasite in hemorrhagic and black-colored lymph nodes were significantly (*P *≤ 0.05) higher than the infection rate in normal-colored nodes. Also, results showed that in soft lymph nodes, the infection rate was significantly (*P* ≤ 0.05) more than those of normal and hard nodes. A high prevalence of infection in camels suggests possibility of similar high rate of infection in other animals, and people in the investigated area. This, in turn, emphasizes the need for more preventive measures to reduce the risk of zoonotic outbreaks.

## Introduction

*Linguatula serrata*, a cosmopolitan parasite, is a member of small group of parasite from phylum Pentastomida.^1^^,^^2^ Adult infects the nasal sinuses and naso-pharynx of carnivorous mammal, especially *Canidae* and probably *Hyaenidae* and *Felidae*.^3^^,^^4^ A wide range of mammals are intermediate hosts for *L. serrata*, but herbivores such as cattle, goats, sheep, camels and other ruminants are the best hosts for development of parasite’s nymphal stages.^5^ Eggs containing fully developed larvae are discharged into the environment by nasal secretion and ingested by the intermediate hosts in which they develops to nymphal stage in the various organs, particularly in Mesenteric Lymph Nodes (MLNs).^6^^-^^8^ Man is occasionally infected with both adult and nymphal stages of *L. serrate*.^9^^-^^11^ Linguatulosis in humans has been reported from various parts of the world, particularly in the Middle-East, Africa, and America and in South-East Asia.^12^^-^^17^

Nasopharyngeal linguatulosis, known also as Halzoun or Marrara syndrome, is the common form of infection in man and is often produced following consumption of raw or undercooked infected viscera (liver, lung and lymph nodes) of infected animals.^14^^,^^18^^,^^19^ This parasite has been reported in people in Iran.^20^^-^^23^ Several studies have been carried out as regards the prevalence of *L. serrata* infection in animals including, dogs,^9^^,^^3^^,^^26^^-^^28^ camels,^29^^-^^33^ buffaloes,^24^^,^^35^ sheep,^35^^-^^39^ cattle,^25^^,^^41^ and goats.^42^^-^^44^

The adult females grow up to 130mm, while males reach only 20mm. They are also flat or annulated and have four hooks surrounding a central mouth.^10^ They keep attached at the wall of the respiratory system by means of their mouth hooks. Females excrete thousands (up to 5,000,000) eggs per day.^10^^,^^24^ These eggs, which contained fully developed larvae, are discharged from the definitive host’s nasopharyngeal secretions and then ingested by the plant feeder animals (including human). Nymphal stages of *L. serrata* in intermediate hosts grow up to 60mm and have four hooks, a mouth, annular rings and spines.^10^^,^^11^^,^^25^ If these nymphs are eaten by the final hosts, the larvae invade the nasal system and reach maturity within 6-7 months and live for about 15 months.^10^

This study was aimed to determine the prevalence of *L. serrata* nymphs in MLNs of camels (*Camelus dromedarius)* slaughtered in Isfahan Province, Central Iran. In addition, in this study morphological characterizations of collected nymphs from camels’ MLNs were analyzed.

## Materials and Methods

Lymph nodes of 232 slaughtered camels at the slaughterhouses of Isfahan Province were examined for *L. serrata *nymphs from April to October 2010. After determining the sex, camels were divided into four age groups (under 6 months, 6-24 months, 2-4 years and more than 4 years) using the eruption of permanent incisor teeth criteria as already described.^45^ At least, three MLNs for each animal and totally 928 nymphs were collected in PBS and transferred to the Parasitology Laboratory of Faculty of Veterinary Medicine of Urmia University. According to Tavassoli *et al*., Lymph nodes were categorized based on their color (normal, red or hemorrhagic and black) and consistency (normal, soft and hard).^37^ After recording the gross appearance, each lymph node was cut longitudinally and examined using a dissecting microscope for *L. serrata* nymphs. The number of collected nymphs from each node was recorded and then nymphs stored in PBS at 4˚C for further studies. The nymphal morphometrics including body length, body width, mouth, hooks, spines and annuli size and some other morphological characterizations were measured using the classic methods of parasitology. In this regard, 50 nymphs were evaluated and their morphological data have been recorded.

The Chi-Square test (SPSS version 17.0) was used to compare the relative frequency of infection among different age and sexes, and among different groups of lymph nodes based on color and consistency. *P* values of equal or less than 0.05 were considered statistically significant. 

## Results

**Fig. 1:**
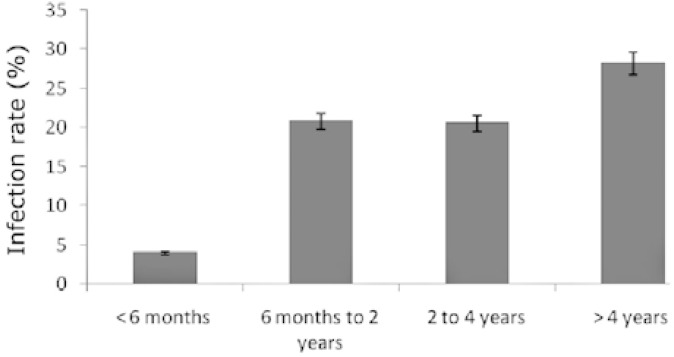
Infection rate of *L. serrata* in different age groups of examined camels.

**Fig. 2:**
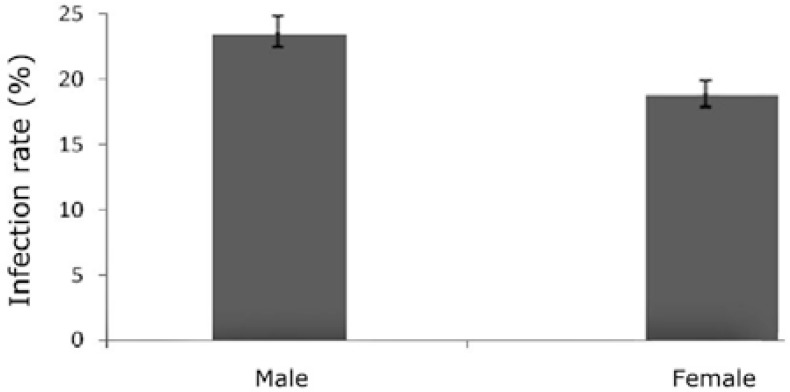
Infection rate of *L. serrata* in male and female animals.

The results showed that MLNs in 49 camels (21.12%) were infected with *L. serrata* nymphs. The number of collected nymphs from each infected lymph node varied from 1 to 29. Table 1 shows the distribution of infection in different age groups and sexes. Figures 1 and 2, show infection rates according to age and sex, respectively. Nymphs appeared tongue-shaped with an anterior swollen body and posterior narrow end. The mean length of nymphs’ body was 4.86 mm (from 3.88 to 5.40 mm) and the mean size of the body width in apical part of nymphs was 1.04 mm (from 0.93 to 1.11 mm). The body width in the end part of nymphs was 0.31 mm at mean (from 0.28 to 0.38 mm). 

**Table 1:** Prevalence rate of *L. serrata* (nymph) in different sex and age groups of camels

**Age**	**Total number**	**Number** **of female animals**	**Number** **of male animals**	**Female positive number**	**Male positive number**
**< 6 months**	25	8	17	0	1
**6-24 months**	53	20	33	4	7
**2-4 years**	83	45	38	10	7
**> 4 years**	71	42	29	13	7

**Fig. 3:**
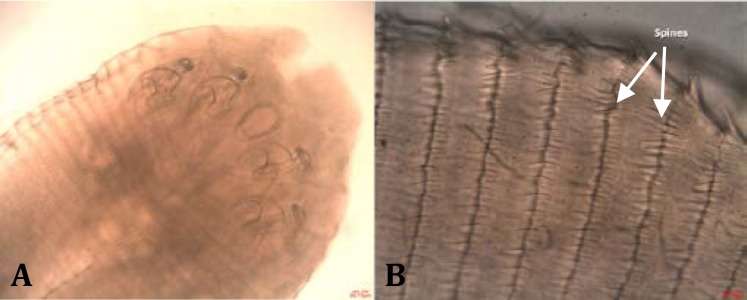
**A:** Mouth and four hooks of *L. serrata* nymphs in anterior part of body (100×) **B:** Arrangement of spines at the posterior edge of each Segment (400×).

**Fig. 4:**
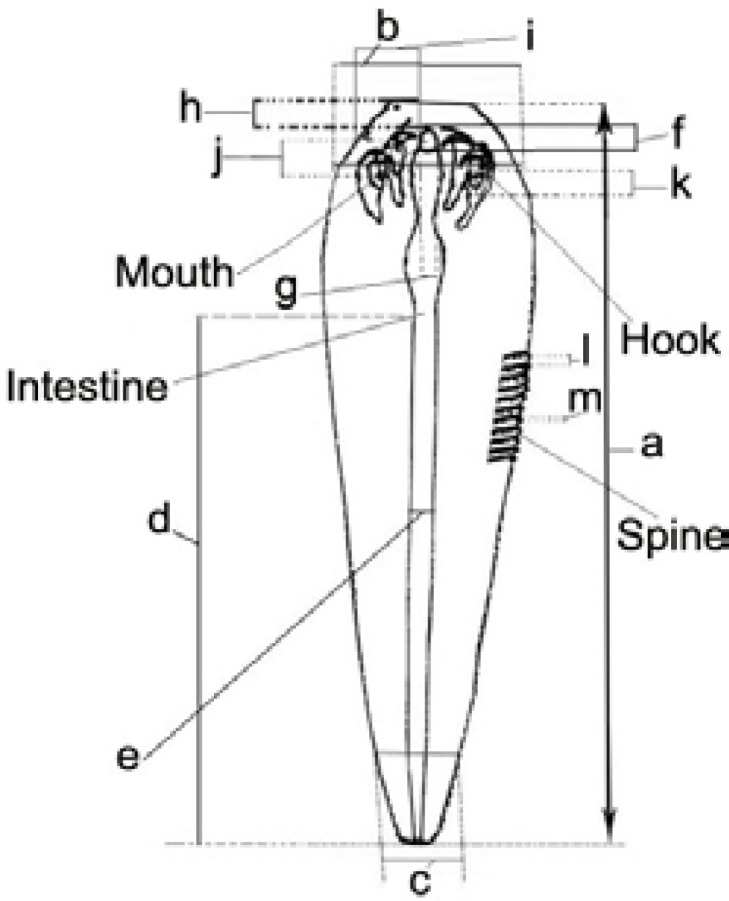
*L. serrata* nymph; **a.** body length, **b.** body width at apex, **c.** body width at base, **d.** intestine length, **e.** intestine width, **f. **mouth length, **g.** mouth width, **h.** distance of mouth from apex,** i.** distance of mouth from margin, **j.** hook width, **k.** hook diameter, **l.** inter segment space, **m.** spine length

The mouth flanked by two pairs of hooks that surrounded by separated suckers (Fig. 3A). Nymphs had segmented body with transversally striated spines and average of 42 per segment, at the posterior edge of each abdominal segment (Fig. 3B). The total number of abdominal segments observed in nymphs was 85. The results of morphometric characterizes of *L. serrata* nymphs are shown in Table 2 and Fig. 4. 

10.40% of normal-colored and 77.60% of hemorrhagic (red) lymph nodes were infected with *L. serrata* nymphs. Also 89.60% of black lymph nodes were infected with nymphs of *L. serrata*. When lymph nodes ranked based on their consistency, 15.60% of normal, 98.00% of soft 29.10% of hard lymph nodes were found infected. Table 3 shows the infection rate of *L. serrata* nymphs in different lymph groups based on color and consistency.

**Table 2:** Morphological data of *L. serrata* nymphs collected from MLNs of camels (50 nymphs evaluated).

**Measured Region**	**Minimum (µm)**	**Maximum (µm)**	**Average (µm)**	**SEM** *
**Body length**	3888.00	5400.00	4869.00	133.63
**Maximum body width**	936.00	1116.00	1044.00	59.45
**Minimum body width**	288.00	381.00	333.90	31.55
**Intestine length**	3060.00	3996.00	3510.33	314.13
**Intestine width**	85.68	99.96	89.25	6.45
**Mouth length**	128.52	171.36	146.37	16.25
**Mouth width**	71.40	86.20	79.50	6.12
**Hook diameter**	108.00	128.52	116.91	5.42
**Hook width**	64.80	86.40	78.24	5.93
**Total hook length**	144.00	162.00	155.40	7.49
**Inter segments space**	39.60	64.80	55.57	10.70
**Distance of mouth from apex**	157.08	199.92	180.88	15.32
**Distance of mouth from margin**	285.60	357.00	304.68	28.77
**Spine length**	28.80	32.40	30.20	1.68
**Spine width**	7.20	9.10	7.90	0.81

* Standard error of the mean.

**Table 3:** Comparison of relative frequency of infection to *L. serrata* lymph nodes categorized based on their color and consistency

**Lymph nodes appearance**	**Number of examined lymph nodes**			**Relative frequency of infection (%)**
	**Total**	**Non-infected**	**Infected**	
**Color** **Normal** **Hemorrhagic** **Black** **Consistency** **Normal** **Soft** **Hard**	747 85 106 772 101 55	669 19 11 651 2 39	78 66 95 121 99 16	10.4 77.6 89.6 15.6 98 29.1

## Discussion

The prevalence of linguatulosis in dogs has been determined in different part of Iran such as 76.20% in Shiraz,^9^ 65.50% in Shahre-Kord,^28^ and 76.47% in Fars province.^26^ In Bursa, Turkey, 20.00% of dogs have been found infected.^46^ Many studies were done on prevalence of *L. serrata* in various domestic ruminants in Iran and other parts of world that *L. serrata* nymphs obtained from different visceral organs such as liver, lung and spleen.^24^^,^^37^^,^^40^^-^^44^ But in most studies MLNs were evaluated because they are the first place of infection with parasite. Therefore, possibility of infection in MLNs is higher than other visceral organs.^33^ Only, few studies regarding the prevalence of *L. serrata* nymphs in camels were conducted in Iran including Mashhad (75.00%)^31^ and Najaf-Abad (35.00% and 21.00%, in two separate studies).^32^^,^^33^ In Egypt 4.90% of MLNs from examined camels were infected.^43^ In recent study, from 232 examined camels, the parasite was found in MLNs of 49 camels, that was higher than the reports of Oryan *et al*. and Wahba *et al*. with infection rate of 7.50% and 4.90%, respectively.^47^^,^^30^ However, our data were less than that reported by Tajik *et al*. and Pourjafar *et al*. with infection rate of 75.00% and 35.00%, respectively.^31^^,^^32^ Therefore, our results showed that Isfahan Province is an endemic area of linguatulosis for camels, and probably for other ruminants and dogs. However, in camels under six months *L. serrata* nymphs only observed in one sample (4.00%). There was not any significant difference among different age groups. Similarly, there was no significant difference in the rate of infection between male and female camels. As camel meat is rather popular in central part of Iran such as Isfahan Province, the high rate of infection with the *L. serrata* nymphs in camels in this area clearly highlights the risk of transmission of the disease through consumption of raw or undercooked camel viscera to human beings. These results also show that infection rate in final hosts such as dogs must be high, and this is the other risk factor for human and ruminants’ linguatulosis. Furthermore, camels offal’s can transmit the infection to the final hosts and cause the life cycle of parasite continue in this area. Based on high prevalence of infection in camels, we supposed that the range of problems associated with linguatulosis in humans is higher than previously thought. Therefore, evaluation of prevalence of infection in human population is needed. In addition, a careful control and treatment strategy are needed for prevention of infections in both intermediate and final hosts of parasite.

In some surveys, morphometric characterizes of *L. serrata* (adult and nymph) and other species of *Linguatula* were investigated. For example, SEM (Scanning Electron Microscope) studies of adult stage of *L. arctica* collected from reindeer and nymphal stage of *L.* *serrata* collected from MLNs of goats were studied.^48^^,^^49^ In other studies, some morphological data of nymphal stage of *Linguatula* *spp. *were measured and recorded. For example the number of abdominal segments was reported to be in the range of 72-97 (mean = 82) in *L. serrata* and 186-232 (mean 210) in *L. multiannulata*. Also, body length of *L. serrata* nymph has been reported to be between 3.40-4.70 mm.^50^ In other study, the number on annuli was 85, body length was between 3.40-4.00 mm and average number of spines in each segment was 42 in collected nymphs from MLNs of slaughtered buffaloes.^34^ Pourjafar *et al*. reported similar data in nymphal stage of *L. serrata* collected from MLNs of slaughtered camels.^32^ In current study, the number of annuli (85) and body length (4.80 mm at mean) were in consistent with previous reports, the other morphological characterizes such as size of mouth, spines, hooks and intestine, the body width in apex and base part of nymphs, were analyzed in our survey (Table 2). However, our results showed that the length of nymphs was more variable (4869.00 ± 133.64 µm) than that of other measured factors in examined nymphs (Table 2). 

Although the greater infection of MLNs, based on their colors and consistencies, found in this study was in comparable with results from previous study that conducted on sheep,^37^ they findings have not been reported in camels. Our results demonstrated that the rate of infection in hemorrhagic and black-colored MLNs was significantly (*P* ≤ 0.05) more than that of in normal-colored ones. However, there was no significant difference (*P* > 0.05) between the infection rate in hemorrhagic and block-colored MLNs. Statistical analysis revealed that the relative frequency of infection in soft lymph nodes was significantly (*P *≤ 0.05) higher than those in normal and hard lymph nodes; and hard lymph nodes were more frequently infected than normal ones (*P* ≤ 0.05). The results from this research suggest that presence of such gross changes in the color and consistency of the MLNs could be considered as an indication of infection of nymphs with *L. serrata* and elimination of such lymph nodes is a necessary step to interrupt the life cycle of parasite.
